# Preparation of Fe_3_O_4_-Embedded Poly(styrene)/Poly(thiophene) Core/Shell Nanoparticles and Their Hydrogel Patterns for Sensor Applications

**DOI:** 10.3390/ma7010195

**Published:** 2014-01-02

**Authors:** Yong Seok Kim, Hyun Jong Lee, Patakamuri Govindaiah, Woohyun Son, Won-Gun Koh, In Woo Cheong, Jung Hyun Kim

**Affiliations:** 1 Department of Chemical and Biomolecular Engineering, Yonsei University, 50 Yonsei-ro, Seodaemoon-Gu, Seoul 120-749, Korea; E-Mails: genie0719@daum.net (Y.S.K.); bml@yonsei.ac.kr (H.J.L.); govindaiah@gmail.com (P.G.); roxxn.son@gmail.com (W.S.); wongun@yonsei.ac.kr (W.-G.K.); 2 Department of Applied Chemistry, Kyungpook National University, 80 Daehakro, Bukgu, Daegu 702-701, Korea

**Keywords:** polythiophene, nanoparticles, fluorescence, sensor, hydrogel patterns

## Abstract

This research describes the preparation and sensor applications of multifunctional monodisperse, Fe_3_O_4_ nanoparticles-embedded poly(styrene)/poly(thiophene) (Fe_3_O_4_-PSt/PTh), core/shell nanoparticles. Monodisperse Fe_3_O_4_-PSt/PTh nanoparticles were prepared by free-radical combination (mini-emulsion/emulsion) polymerization for Fe_3_O_4_-PSt core and oxidative seeded emulsion polymerization for PTh shell in the presence of FeCl_3_/H_2_O_2_ as a redox catalyst, respectively. For applicability of Fe_3_O_4_-PSt/PTh as sensors, Fe_3_O_4_-PSt/PTh-immobilized poly(ethylene glycol) (PEG)-based hydrogels were fabricated by photolithography. The hydrogel patterns showed a good sensing performance under different H_2_O_2_ concentrations. They also showed a quenching sensitivity of 1 μg/mL for the Pd^2+^ metal ion within 1 min. The hydrogel micropatterns not only provide a fast water uptake property but also suggest the feasibility of both H_2_O_2_ and Pd^2+^ detection.

## 1. Introduction

Recently optical sensing materials have received great attention and these can be categorized in two groups, organic and inorganic. One typical of the inorganic material group is quantum dots (QDs) [1], however, several disadvantages have emerged such as short fluorescence lifetime, susceptibility to photo-bleaching, and complexity in fabrication processes requiring multiple steps which also makes the production costly. Alternatively, conjugated polymers from the organic material group are widely used for various sensing applications. Among these, poly(thiophene) (PTh) and its derivatives have received great attention as sensing materials, particularly for DNA [2], gas [3], glucose oxidase (GOX) [4], ion chromatism [5], magnetometer [6] and pH [7]. In previous studies, we presented a synthetic mechanism of pristine PTh nanoparticles [8], a size controlling process [8], a kinetic mechanism of PSt/PTh core/shell nanoparticles [9], the control of fluorescence properties by varying the PTh shell thickness of the PSt/PTh core/shell nanoparticles [10], and a new composite mechanism and properties of hybrid Fe_3_O_4_ NPs-PSt/PTh composite nanoparticles [11].

As part of a sensing system, hydrogels of a 3D network structure can effectively enhance sensitivity, *i.e.*, the ratio of signal to noise, owing to the swellability for large capacity and permeability of the targeting medium as well as a dimensional difference compared to a 2D sensory surface. Among hydrogels, poly(ethylene glycol) (PEG)-based hydrogels have been widely used to immobilize biomolecules or organic materials due to high water holding capacity, hydrophilicity and biocompatibility [12–21]. In addition, PEG-based hydrogels can easily be modified by manipulating the molecular weight, the type and degree of crosslink, and the dimensions from micro- to millimeter-scale for versatility [22].

This work describes the preparation of monodisperse Fe_3_O_4_-PSt/PTh core/shell nanoparticles and immobilization of the nanoparticles into the crosslinked PEGDA hydrogel matrix via photolithography for the detection of H_2_O_2_ as well as Pd^2+^ and Fe^3+^ as a function of concentration with fast response detection time. As a result, the multifunctional sensing performance, properties and the versatile patterning process of nanoparticles in the hydrogel matrix as a new sensing nanomaterial make them highly suitable candidates for sensors.

## 2. Results and Discussion

**Figure 1.**
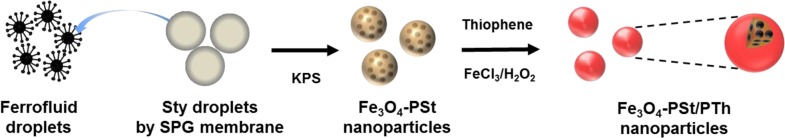
A schematic illustration of the preparation of Fe_3_O_4_ nanoparticles-embedded poly(styrene)/poly(thiophene (Fe_3_O_4_-PSt/PTh) core/shell nanoparticles.

A schematic diagram of monodisperse Fe_3_O_4_-PSt/PTh core/shell nanoparticles is illustrated in Figure 1. Oleic acid-stabilized Fe_3_O_4_ nanoparticles were prepared by a co-precipitation method and dispersed into octane to form the ferrofluid. On the other hand, styrene droplets were prepared by membrane emulsification and the resulting droplets (~3 μm in diameter) are shown in the supporting information (Figure S1). Monodisperse Fe_3_O_4_ NPs-PSt nanoparticles were prepared through the so-called “activated swelling method” of emulsion polymerization [23] where the smaller ferrofluid droplets are polymerization loci. Styrene monomer was replenished by micron-sized styrene monomer droplets. These can be rationalized by the average particle size of the resulting Fe_3_O_4_-PSt nanoparticles, as shown in Figure S2a. A polythiophene shell was then formed by oxidative polymerization in the presence of FeCl_3_/H_2_O_2_ [11].

**Figure 2.**
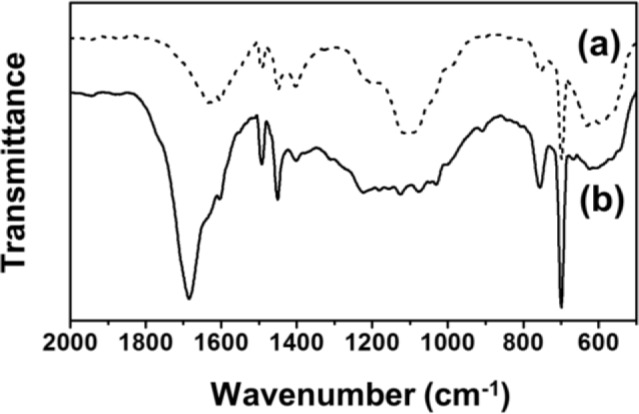
Fourier transform-infrared (FT-IR) spectra of (**a**) Fe_3_O_4_-PSt and (**b**) Fe_3_O_4_-PSt/PTh core/shell nanoparticles.

For comparison, the FT-IR spectra of Fe_3_O_4_-PSt and Fe_3_O_4_-PSt/PTh core/shell nanoparticles are shown in Figure 2. The vibration band at 580 cm^−1^ was assigned to the Fe-O of the iron oxide, which confirmed the presence of the Fe_3_O_4_ nanoparticles in both Fe_3_O_4_-PSt and Fe_3_O_4_-PSt/PTh core/shell nanoparticles. The C–H bond of PSt was found at 1450 cm^−1^ and the strong absorption bands at 1470 and 1590 cm^−1^ were attributed to the stretching vibration of C=C bond on the benzene ring. The sharp bands 700 and 750 cm^−1^ were assigned to the out-of-plane bending vibrations of the mono-substituted benzene ring. After the oxidative polymerization, the strong absorption band at 1690 cm^−1^ newly appeared, indicating the stretching vibration of the C=C bond from PTh. These data confirmed the PTh shell layer formation.

Figure S3 shows the TGA curves of the PSt and Fe_3_O_4_-PSt nanoparticles. For PSt, the initial decomposition of SDS, OA were found at 210–240 °C in Figure S3b [24]. The weight loss of about 20% at 400 °C in Figure S3b is the typical burn out temperature of PSt in Figure S3a. Importantly, the TGA curves revealed that ca. 69 wt% Fe_3_O_4_ nanoparticles were encapsulated in the nanoparticles.

**Figure 3.**
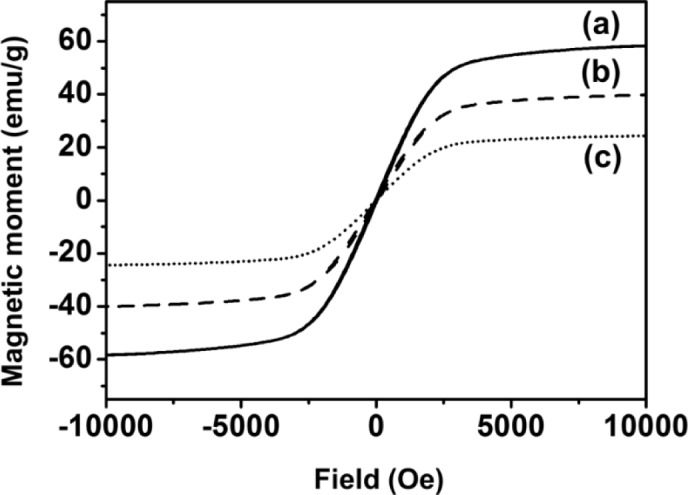
Magnetization hysteresis loops of (**a**) Fe_3_O_4_; (**b**) Fe_3_O_4_-PSt and (**c**) Fe_3_O_4_-PSt/PTh core/shell nanoparticles measured by superconducting quantum interference device (SQUID) at room temperature.

As shown in Figure 3, the magnetic properties of Fe_3_O_4_, Fe_3_O_4_-PSt and Fe_3_O_4_-PSt/PTh core/shell nanoparticles were studied with SQUID magnetometry at room temperature as a function of external field (−1 to 1 Tesla). The zero coercivity and the reversible hysteresis behavior indicate the superparamagnetic nature of the nanoparticles. The saturation magnetization value of Fe_3_O_4_ nanoparticle was 63 emu/g at 300 K. In addition, the magnetic moment of Fe_3_O_4_-PSt nanoparticles was higher than that in the previous report due to the higher volume fraction of magnetite in the nanoscale (<100 nm) [11]. After PTh (~3 nm thickness) sheath formation, the magnetic moment was decreased from 43 emu/g to 25 emu/g. This seems mainly attributable to not only the possible electromagnetic shielding effect of conjugated polymer but also to the decrease of the magnetite content and the magnetically dead layer on the shell of the particles or to a spin glass-like behavior of the surface of spins or canted spins [11].

**Figure 4.**
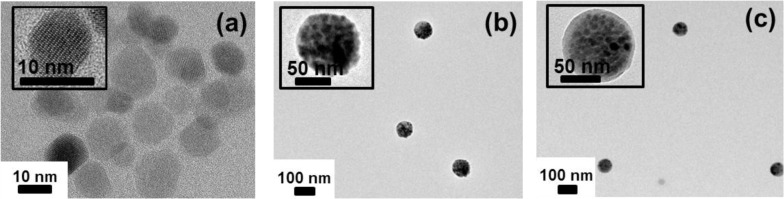
Transmission electron microscopy (TEM) micrographs of (**a**) oleic acid-stabilized Fe_3_O_4_ nanoparticles; (**b**) Fe_3_O_4_-PSt and (**c**) Fe_3_O_4_-PSt/PTh core/shell nanoparticles. The insets of micrographs are (**a**) HR-TEM (**b**,**c**) highly magnified images of the corresponding sample.

Figure 4 shows the TEM micrographs of oleic acid-stabilized Fe_3_O_4_ nanoparticle, Fe_3_O_4_-PSt, and Fe_3_O_4_-PSt/PTh nanoparticles. As can be seen in the inset of Figure 4a, the lattice fringes in the HR-TEM image correspond to a group of atomic planes within a single crystal of Fe_3_O_4_ nanoparticle. As shown in Figure 4b, the ${\bar{D}}_{z}$ of Fe_3_O_4_-PSt nanoparticles was 84.9 nm and the PDI was 0.048 (fairly monodisperse). The Fe_3_O_4_-PSt nanoparticle seems to be fully filled with Fe_3_O_4_ nanoparticles. Figure 4c clearly shows the bright shell layer of PTh. In addition, the ${\bar{D}}_{z}$ of Fe_3_O_4_-PSt/PTh nanoparticles was increased to 87.3 nm (PDI = 0.061). These results confirm that Fe_3_O_4_-PSt/PTh nanoparticles were successfully prepared.

**Figure 5.**
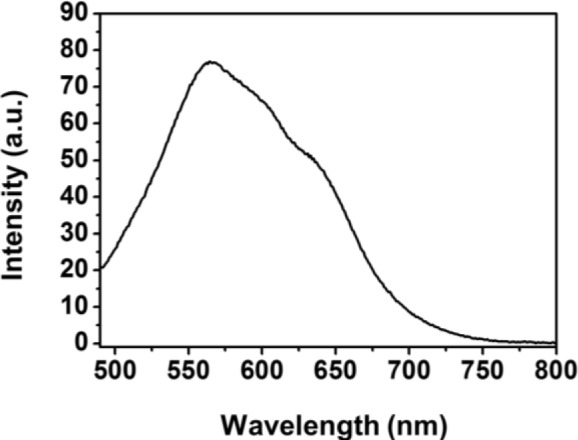
Photoluminescence (PL) spectra of Fe_3_O_4_-PSt/PTh core/shell particles in an emulsion state (0.009 wt%) at an excitation wavelength of 400 nm.

The PL spectra of Fe_3_O_4_-PSt/PTh core/shell nanoparticles were measured by spectrofluorophotometry at an excitation wavelength of 400 nm and plotted in Figure 5. The maximum emission wavelength (λ_max_) was 564 nm with red emission in previous report. Similarly, Fe_3_O_4_-PSt nanoparticles with the thin PTh shell also emitted red light. Also, the Fe_3_O_4_ NPs-embedded core part could not quench the PL emission [11].

**Figure 6.**
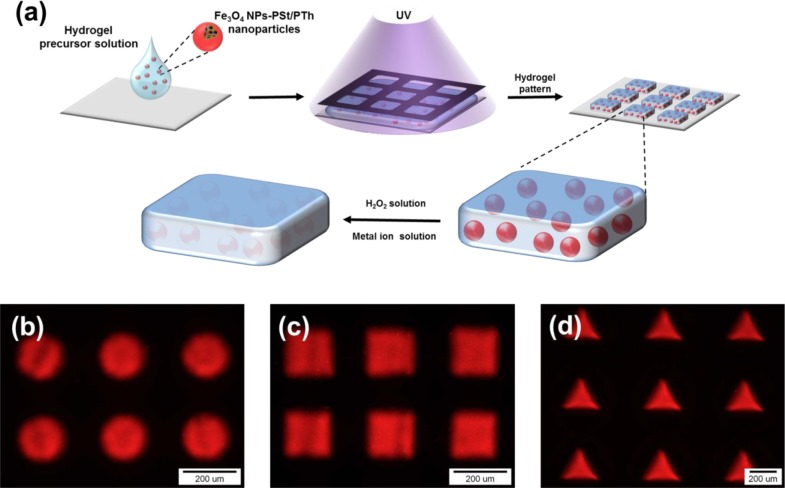
(**a**) A schematic illustration for the fabrication of hydrogel patterns by photolithography. The fluorescent emission from the Fe_3_O_4_-PSt/PTh is quenched by H_2_O_2_ or metal ion solution; (**b**–**d**) fluorescence microscopic images of Fe_3_O_4_-PSt/PTh nanoparticle-immobilized hydrogel patterns with different shapes: (**b**) circle; (**c**) square and (**d**) triangular shapes.

For sensor application, Fe_3_O_4_-PSt/PTh core/shell nanoparticles were immobilized in the hydrogel patterns, as shown in Figure 6a. The sizes and shapes of the hydrogel patterns were regulated by photomasks in the lithography process, and the patterns of circle, square, and triangle shapes were fabricated. This multiple shape hydrogel particle could be also utilized for multiple analyses. It would be possible to detect other modified Fe_3_O_4_ NPs-PSt/PTh nanoparticles which have each different properties using a shape-coded detection system as presented previously [13,18,19].

In order to study the concentration effect of Fe_3_O_4_-PSt/PTh nanoparticles, the fluorescence intensity of the hydrogel was recorded at the different nanoparticle concentrations of 5 and 10 wt%. As shown in Figure S4, the uniformity of fluorescence intensity from the 5 wt% Fe_3_O_4_-PSt/PTh sample was more even than the inhomogeneity of 10 wt%. The results were attributed to the fluorescence uniformity originating from the aggregation of Fe_3_O_4_-PSt/PTh nanoparticles in the hydrogel matrix owing to the hydrophobic nature of the nanoparticles against the hydrophilic nature of the hydrogel matrix. Thus, applicability of Fe_3_O_4_-PSt/PTh nanoparticles as a sensor was evaluated using 200 × 200 μm^2^ squared hydrogel patterns at the predetermined nanoparticle concentration of 5 wt%.

**Figure 7.**
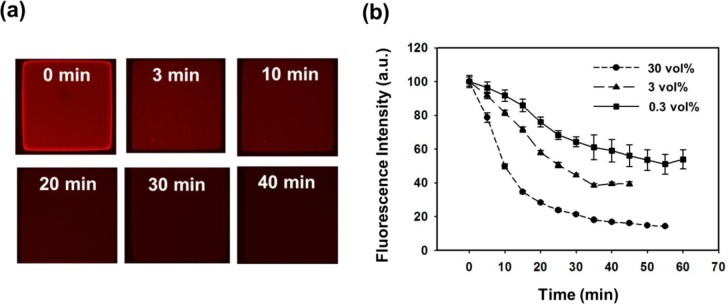
(**a**) Fluorescence micrographs of Fe_3_O_4_-PSt/PTh immobilized hydrogel patterns in the presence of 30 vol% H_2_O_2_ with different times and (**b**) variations of the fluorescence intensity as a function of contact time with different H_2_O_2_ concentration.

Various oxidase produce H_2_O_2_ during the catalytic oxidation-reduction in the presence of oxygen [12–14,20,21]. As a result, H_2_O_2_ quenches the photoluminescence of the fluorophore by electron transfer [25]. As for H_2_O_2_ sensing feasibility, the fluorescence images were recorded and the data for the square hydrogel patterns are summarized in Figure 7. The fluorescence intensity in Figure 7b decreased with the lapse of time. As the concentration of H_2_O_2_ decreased, the slope of fluorescence intensity became less steep. With 30 vol% H_2_O_2_, the intensity steeply decreased to 20% of the initial intensity within 35 min, and gradually decreased until 55 min. As for 3 vol% H_2_O_2_, the intensity was decreased to 40% when the contact time was 35 min. The rate of fluorescence quenching increased with the concentration of H_2_O_2_. The mechanism relied on the electron-transfer reaction that occurred at the surface of the Fe_3_O_4_-PSt/PTh nanoparticles where H_2_O_2_ was reduced to O_2_, which in turn lay in electron/hole traps on the polythiophene and could be used as a good electron acceptor, thus forming the non-fluorescent Fe_3_O_4_-PSt/PTh nanoparticles and leading to reduced fluorescence.

**Figure 8.**
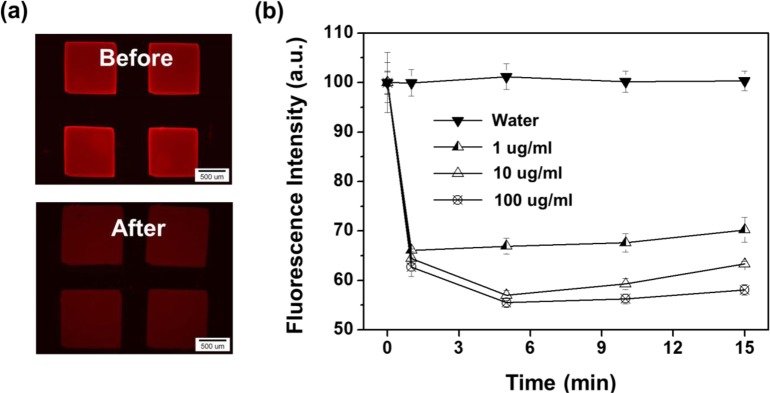
(**a**) Fluorescence micrographs of Fe_3_O_4_-PSt/PTh immobilized hydrogel pattern in the 100 μg/mL Pd^2+^ and (**b**) variations of the fluorescence intensity as a function of contact time with different Pd^2+^ concentrations.

Regarding the feasibility test for metal ion detection, the fluorescence intensities of Fe_3_O_4_-PSt/PTh immobilized hydrogel patterns in Pd^2+^ ion solution were recorded and the data are shown in Figure 8. Figure 8a shows the fluorescence images of the hydrogel patterns in DDI water (before) and 100 μg/mL Pd^2+^ aqueous solution (after), respectively. As shown in Figure 8b, the fluorescence intensities decreased by 35% for all concentrations from 1 μg/mL to 100 μg/mL of Pd^2+^ ions within 1 min. The concentration dependency on the variation in fluorescence intensity was observed after several minutes. Fe^3+^ ion was also tested with 10 μg/mL Fe^3+^ aqueous solution, as shown in Figure S5, and the data presented a similar result with Pd^2+^ ion. About 20% of fluorescence intensity decreased immediately, and the intensity dropped until 70% of initial intensity over 5 min. Compared with the same concentration of Pd ion, the Pd ion decreased the fluorescence more dramatically than the iron ion because of a property against the oxidative nature of Fe^3+^ under acidic conditions and the electro-active nature of polythiophene due to non-acidic conditions. Also, Pd^2+^ might interact with sulfur of the PTh shell via chelating or complex reactions [26–28]. In addition, complex formation of palladium ion (as a soft acid) with PTh (as a soft base) can be considered as a type of Lewis acid/base (termed as soft acid–soft base reactions) might be a possible description for higher sensitivity of nanoparticles toward palladium ion.

## 3. Experimental Section

### 3.1. Materials

All reagents used in this work were analytic grade and available commercially. Ferric chloride (FeCl_3_·6H_2_O), ferrous chloride (FeCl_2_·4H_2_O), hexadecane (99%), styrene (St), thiophene (Th), Poly(ethylene glycol) diacrylate (PEGDA, M.W. = 575 g/mol), 2-hydroxy-2-methylpropiophenone (HOMPP, 97%) were purchased from Sigma-Aldrich (St. Louis, MA, USA). Ammonia water (NH_4_OH, 30%, Duksan Pure Chemicals Co., Ltd., Ansan-si, Korea), sodium dodecyl sulfate (SDS, Extra Pure, Duksan Pure Chemicals Co., Ltd.), poly(oxyethylene) (20) sorbitan monolaurate (Tween 20, Duksan Pure Chemicals Co., Ltd.), and potassium persulfate (KPS, Junsei Chemical, Tokyo, Japan) were used without purification. Anhydrous FeCl_3_ (Kanto Chemical, Tokyo, Japan) and hydrogen peroxide (H_2_O_2_, 30%, Duksan Pure Chemicals Co., Ltd.) were used as an oxidant and reductant, respectively, without further purification. Double-distilled and deionized (DDI) water was used throughout the experiments.

### 3.2. Preparation of Monodisperse Fe_3_O_4_-PSt/PTh Core/Shell Nanoparticles

Fe_3_O_4_-embedded PSt nanoparticles (Fe_3_O_4_-PSt) were prepared by combined mini-emulsion/emulsion polymerization as reported elsewhere [23]. For the preparation of Fe_3_O_4_ nanoparticle, FeCl_3_·6H_2_O (24 g) and FeCl_2_·4H_2_O (10 g) were dissolved in DDI water (100 mL) at 80 °C under N_2_ atmosphere with vigorous stirring. Ammonia water (50 mL) was then quickly added into the above solution and the solution turned black, which was a sign of forming a black precipitate. For the hydrophobic modification of Fe_3_O_4_, oleic acid (3.76 g) was added dropwise for 20 min, at 80 °C for 1 h, and then ultra-sonicated for 15 min. The oleic acid-coated Fe_3_O_4_ nanoparticles were subsequently collected and transferred into octane to make a ferrofluid with a magnetite content of 68 wt%.

A porous glass membrane (SPG membrane, pore size = 1.0 μm, SPG Technology Co. Ltd., Miyazaki, Japan) was used as a template for the mini-emulsion polymerization of styrene. Styrene (2.5 g) and hexadecane (0.04 g) were mixed and added into the vessel for dispersion phase. SDS (0.05 g) was then dissolved in water (40 mL) for the continuous phase. Monodisperse styrene droplets were obtained under the administration of 38 kPa pressure (N_2_ gas) for 3 h. In order to introduce Fe_3_O_4_ nanoparticles, the ferrofluid (Fe_3_O_4_, 2 g, 68 wt% in octane) was added into the SDS aqueous solution (0.05 g SDS in 48 mL DDI water) and this mixture was ultra-sonicated (500 W, VCX-750, Sonic Inc., Atlanta, GA, USA) in an ice bath for 15 min.

For the preparation of Fe_3_O_4_-PSt, the above emulsions were added into a three-neck round-bottomed flask in the presence of KPS (20 mg) and stirred for 30 min under N_2_ atmosphere. Then the reactor was placed in a water bath at 80 °C to initiate polymerization. The reaction was carried out for 24 h. The resulting Fe_3_O_4_-PSt was collected by using a permanent magnet for purification. The z-average particle size ( ${\bar{D}}_{z}$) of Fe_3_O_4_-PSt was 84.9 nm and the polydispersity index (PDI) was 0.048, measured by dynamic light scattering.

For the preparation of Fe_3_O_4_-PSt/PTh core/shell nanoparticles, Fe_3_O_4_-PSt (1.5 g) was redispersed in DDI water (45 g) by bath sonication in the presence of Tween 20 (0.5 g). Above Fe_3_O_4_-PSt emulsion was stirred for 10 min for stabilization at room temperature and subsequently, thiophene monomer (0.5 g) and H_2_O_2_ aqueous solution (1 g) were added after another 10 min in order. After 10 min, FeCl_3_ (0.005 g) dissolved in DDI water (5 g) was slowly added to initiate polymerization. The polymerization was kept for 24 h. Finally, monodisperse Fe_3_O_4_-PSt/PTh core/shell particles were obtained with ${\bar{D}}_{z}$ of 87.3 nm (PDI = 0.061). The yield of the product was 91% ± 4% after purification.

### 3.3. Fabrication of Hydrogel Patterns

The hydrogel patterns were fabricated via photolithography. PEGDA was first mixed with Fe_3_O_4_-PSt/PTh emulsion (5 wt%) as a 1:1 volume ratio, and then 2 vol% of HOMPP was added to the mixture. After 50 μL of precursor solution was dropped onto a slide glass, and 365 nm (10 mW/cm^2^) UV light (EFOS Ultra-cure 100ss Plus, UV sport lamp, Mississauga, ON, Canada) was applied through photomasks for 1 s. Unreacted precursor solution was removed via development process with water.

### 3.4. Characterization

The average particle size and morphology of Fe_3_O_4_-PSt/PTh nanoparticles were measured by dynamic light scattering (DLS, Zetasizer Nano ZS, Malvern, PA, USA), field emission scanning electron microscopy/energy dispersive spectroscopy (FE-SEM/EDS, JSM-6701F, JEOL Ltd., Tokyo Japan), and transmission electron microscopy (TEM, JEM-2000EXII, JEOL Ltd.). Fourier transform-infrared spectra were recorded by FT-IR (Tensor 27, Bruker Corp., Rheinstetten, Germany) in KBr pellets. All samples were scanned in the range 510–2000 cm^−1^. Photoluminescence (PL) spectra were recorded in a spectrofluorophotometer (RF-5301PC, Shimadzu, Kyoto, Japan). The thermal properties of the nanoparticles were analyzed by thermogravimetry (TGA) (Q50, TA Instr., New Castle, DE, USA). Magnetization hysteresis loops were measured with a superconducting quantum interference device (SQUID) magnetometer (MPMS XL, Quantum Design, Inc., lan Diego, CA, USA) at room temperature. The saturation magnetization values were normalized to the mass of nanoparticles to yield the specific magnetization, M (emu/g). A Zeiss Axiovert 200 microscope equipped with an integrated color CCD camera (Carl Zeiss Inc., Thornwood, NY, USA) was used to obtain the fluorescence images of the fluorescent hydrogel microstructures. Image analyses were performed using commercially available image analysis software (KS 300, Carl Zeiss Inc., Oberkochen, Germany).

## 4. Conclusions

We synthesized monodisperse Fe_3_O_4_-PSt/PTh core/shell nanoparticles successfully by combinational mini-emulsion/emulsion polymerization and oxidative polymerization. TEM and TGA results indicated that the high concentration (69 wt%) of Fe_3_O_4_ nanoparticles were embedded in the monodisperse PSt matrix even after PTh sheath formation during the oxidative polymerization. The Fe_3_O_4_-PSt/PTh nanoparticles were then immobilized in crosslinked PEG hydrogel patterns for their applicability as sensors, and this was based on the quenching effect of H_2_O_2_ or Pd^2+^ ion. The variations in fluorescence intensity arising from H_2_O_2_ and the metal ion were evaluated with different concentrations and contact times. The results suggest that the Fe_3_O_4_-PSt/PTh nanoparticle could be a candidate for fluorescence quenching-based optical sensors providing a size and shape controllable multiplex detection system.

## Supplementary Materials

Supplementary File 1
