# Supplementary material for: Preparation of Fe3O4-Embedded Poly(styrene)/Poly(thiophene) Core/Shell Nanoparticles and Their Hydrogel Patterns for Sensor Applications
Source: Materials (Basel). 2014 Jan 2;7(1):195–205. doi: 10.3390/ma7010195 (PMC5453122; doi:10.3390/ma7010195)

Preparation of Fe_3O_4-Embedded Poly(styrene)/Poly(thiophene) Core/Shell Nanoparticles and Their Hydrogel Patterns for Sensor Applications

Figure S1. Optical micrograph of styrene droplets prepared by using SPG membrane emulsification.

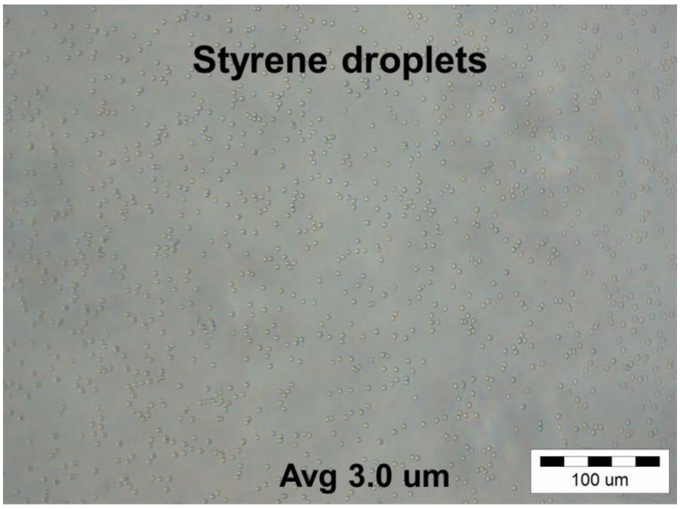

Figure S2. Particle size distributions of (**a**) Fe_3O_4-PSt and (**b**) Fe_3O_4-PSt/PTh nanoparticles dispersed in DDI water at the same concentration of about 0.01 wt%.

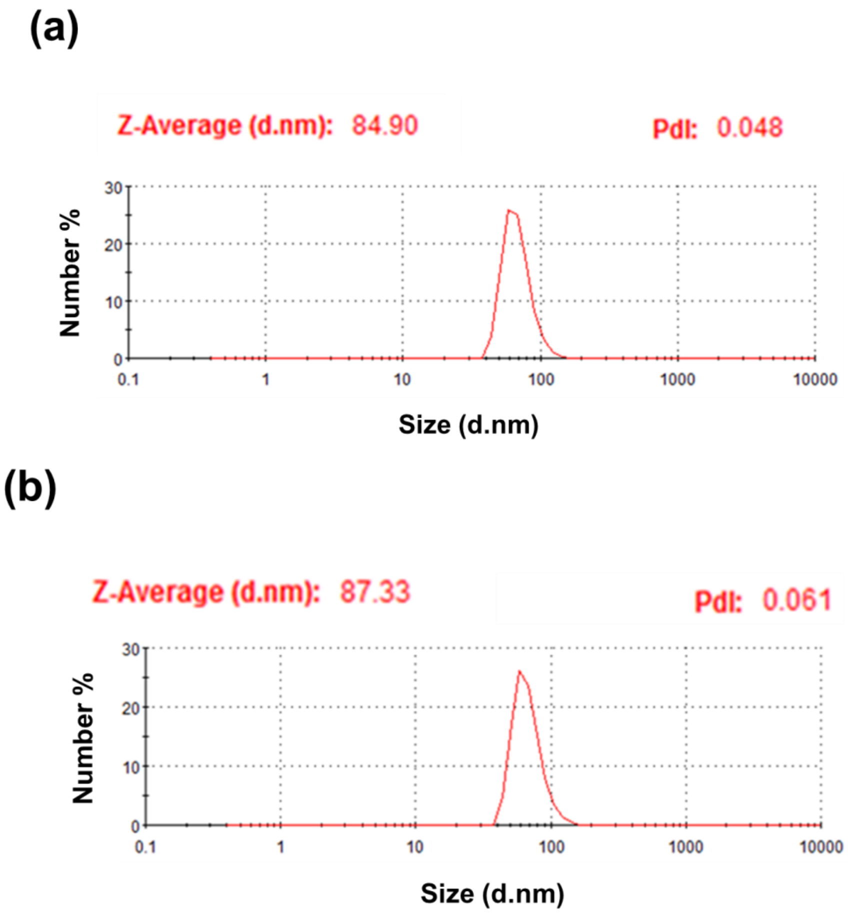

Figure S3. TGA curves of (**a**) as-synthesized PSt and (**b**) Fe_3O_4-PSt nanoparticles.

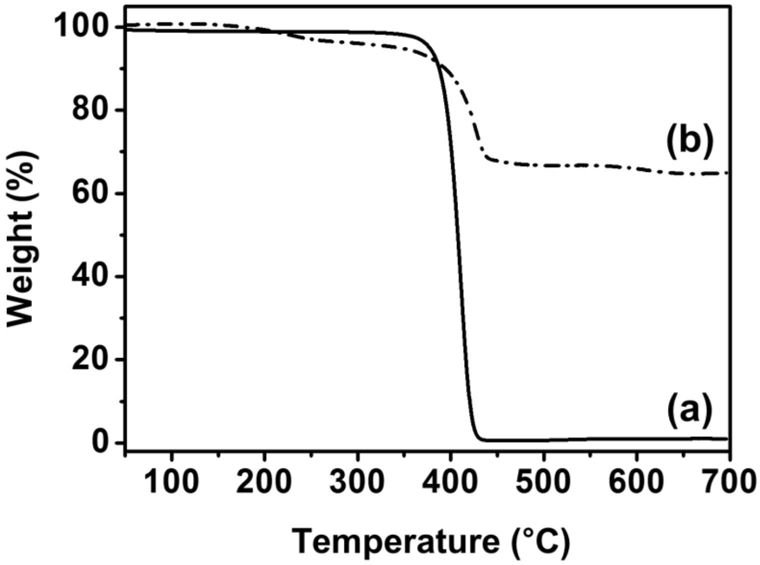

Figure S4. Fluorescence images of Fe_3O_4-PSt/PTh nanoparticles-immobilized hydrogel patterns, (**a**) 10 wt% and (**b**) 5 wt% Fe_3O_4-PSt/PTh nanoparticle concentrations, respectively. (**c**) is corresponding profile of fluorescence intensities recorded at the dashed lines in (**a**) and (**b**).

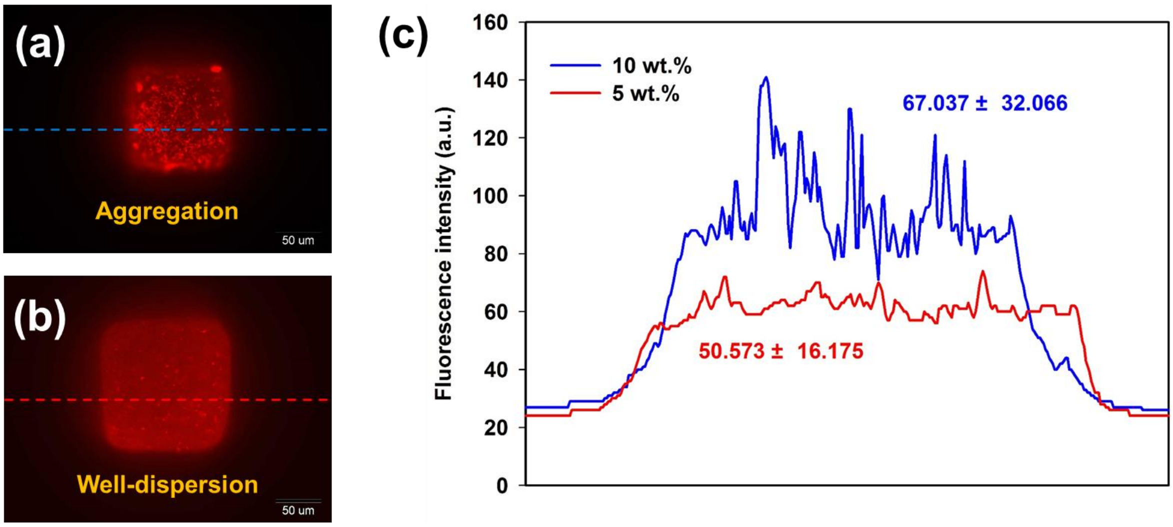

Figure S5. (**a**) Fluorescence micrographs of Fe_3O_4-PSt/PTh immobilized hydrogel patterns in the 10 μg/mL Fe^{3+} aqueous solution and (**b**) corresponding fluorescence intensity as a function of contact time.

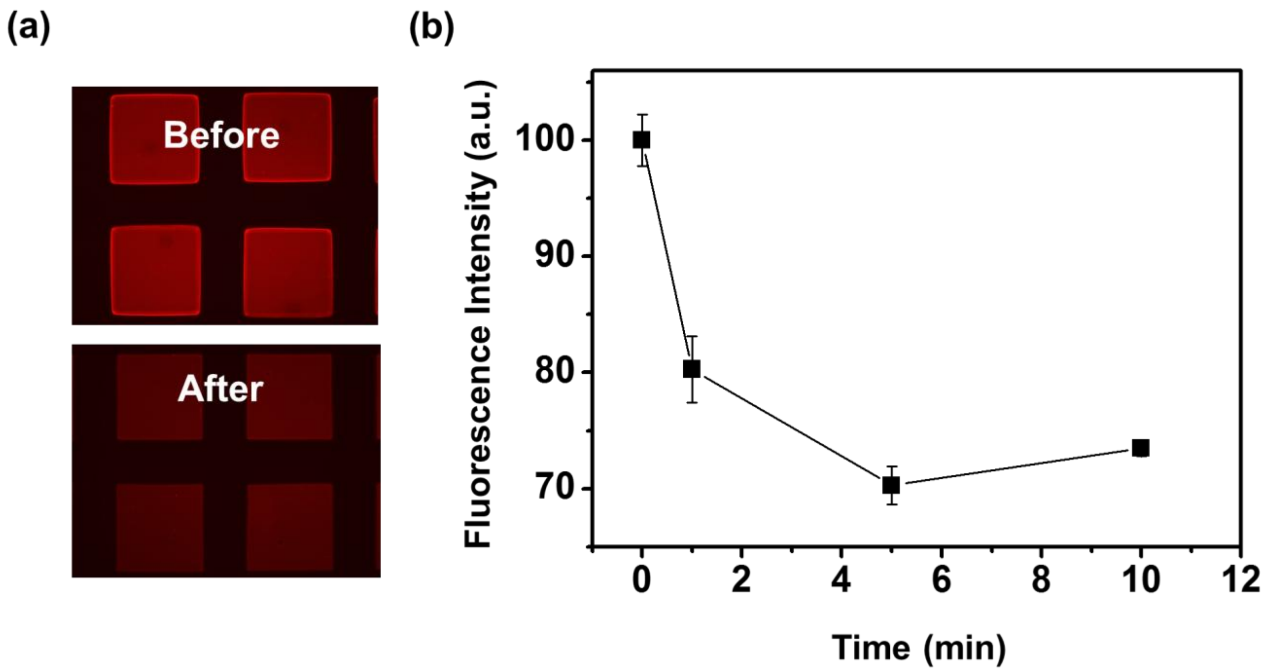

Supplement: Supplementary File 1 [file materials-07-00195-s001.pdf]
